# P-1340. Genetic Factors Associated with Cefepime and Piperacillin/Tazobactam Resistance in Clinical Extended-Spectrum Beta-Lactamase-Producing Enterobacterales from the Intensive Care Unit

**DOI:** 10.1093/ofid/ofae631.1517

**Published:** 2025-01-29

**Authors:** Todd W Hokunson, Erica Hernandez, Medini K Annavajhala, Anne-Catrin Uhlemann, Angela Gomez-Simmonds

**Affiliations:** Columbia University Medical Center, New York, New York; Albert Einstein College of Medicine, Bronx, New York; Columbia University, New York, New York; Columbia University Irving Medical Center, New York, NY; University of California, Davis, Sacramento, California

## Abstract

**Background:**

Extended-spectrum β-lactamase (ESBL)-producing Enterobacterales (ESBLE) are common pathogens in intensive care units (ICUs) causing infection at a range of body sites. While carbapenems are considered first-line agents, non-carbapenem beta-lactams such as piperacillin/tazobactam (TZP) and cefepime (FEP) are often used to treat non-severe ESBLE infections. Few studies have examined the molecular epidemiology and mechanisms of TZP and FEP resistance in non-bloodstream ESBLE isolates (e.g. urine, respiratory tract).

**Figure:**
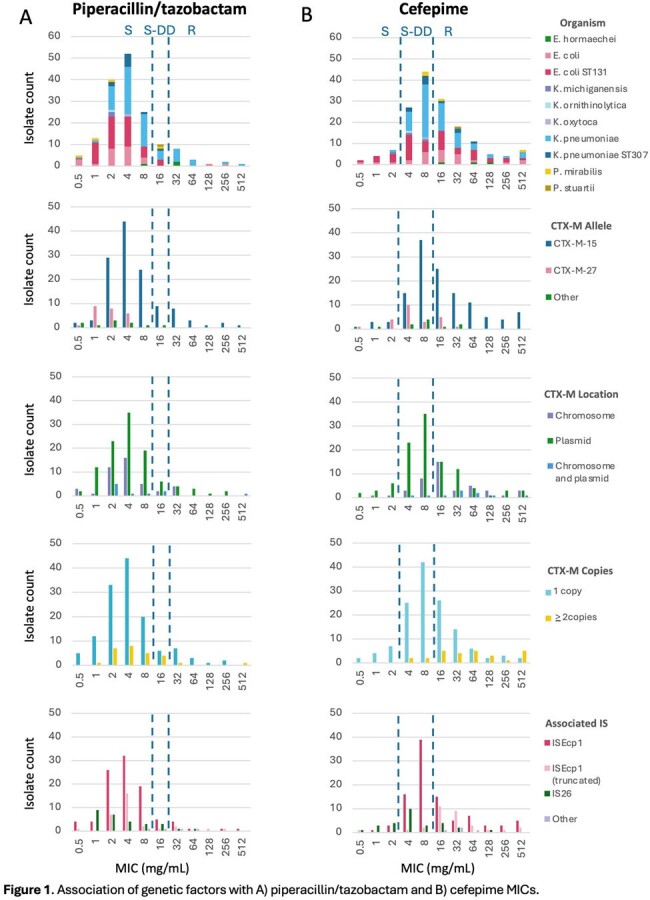

**Methods:**

We screened Enterobacterales isolates collected between 2015-2019 for the ESBL gene *bla*_CTX-M_ using multiplex PCR and performed nanopore sequencing positive isolates (n=164). FEP and TZP susceptibilities were determined by broth microdilution (BMD). We assessed the impact of genetic factors such as the location and genetic context of *bla*_CTX-M_, other beta-lactamase gene presence, and porin gene disruption on TZP and FEP MICs (**Figure 1**); univariable and multivariable logistic regression were used to test their association with FEP and TZP resistance.

**Results:**

Most isolates belonged to *Klebsiella pneumoniae* (47%) or *Escherichia coli*(45%) and were highly clonally diverse. *bla*_CTX-M-15_ was the most common *bla*_CTX-M_ allele (78%) followed by *bla*_CTX-M-27_ (15%). 47% of isolates demonstrated FEP resistance (MIC50/90 8/64 µg/mL), which was significantly associated with *bla*_CTX-M_ allele, location, and copy number and narrow-spectrum β-lactamase co-carriage. Only 10% of isolates were resistant to TZP (MIC50/90 4/16 µg/mL), which did not appear to be modulated by presence of *bla*_CTX-M_. In multivariable analyses, FEP resistance was independently associated with *bla*_CTX-M_ copies (OR 5.21, 95% CI 1.7-24.6, *p*=0.01) and association with truncated IS*Ecp1* (OR 12.9, 95% CI 3.1-65, *p*< 0.001); TZP resistance was associated with organism (OR 5.82 for *K. pneumoniae* versus *E. coli*, 95% CI 1.3-43.8, *p*=0.04), presence of *bla*_OXA-1_ (OR 5.2, 95% CI 1.3-35.9, *p*=0.04), and porin gene disruption (OR 6.5, 95% CI 1.3-32.3, *p*=0.02).

**Conclusion:**

In summary, TZP resistance was uncommon and appeared to be independent of *bla*_CTX-M_ carriage. TZP may be an appropriate treatment for nonbloodstream ESBLE infections in the ICU and should be evaluated further in clinical studies.

**Disclosures:**

**All Authors**: No reported disclosures

